# Case report: Five-year periodontal management of a patient with two novel mutation sites in *ELANE*-induced cyclic neutropenia

**DOI:** 10.3389/fgene.2022.972598

**Published:** 2022-11-01

**Authors:** Zhentao Lao, Jiarun Fu, Zhiying Wu, Lihong Zhu, Shiwen Wu, Yongheng Lin, Chaoming Hu, Dingyu Duan, Panpan Wang

**Affiliations:** ^1^ Guangdong Provincial Key Laboratory of Stomatology, Guanghua School of Stomatology, Sun Yat-Sen University, Guangzhou, Guangdong, China; ^2^ Department of Microbiology, Key Laboratory for Tropical Diseases Control of the Ministry of Education, Zhongshan School of Medicine, Sun Yat-sen University, Guangzhou, Guangdong, China; ^3^ Department of Stomatology, The Second Clinical Hospital of Jinan University, Shenzhen People’s Hospital, Shenzhen, China; ^4^ Institute of Stomatology, School and Hospital of Stomatology, Wenzhou Medical University, Wenzhou, China; ^5^ State Key Laboratory of Oral Diseases, Department of Periodontology, National Clinical Research Center for Oral Diseases, West China School and Hospital of Stomatology, Sichuan University, Chengdu, China; ^6^ Department of Periodontology, Guanghua School and Hospital of Stomatology, Sun Yat-sen University, Guangzhou, Guangdong, China

**Keywords:** cyclic neutropenia, *ELANE* gene, periodontitis, tooth displacement, oral manifestation, case report

## Abstract

Cyclic neutropenia (CyN) is a rare, *ELANE*-related neutropenia. Oral manifestations are among the initial signs of CyN and an important reason that leads patients to seek professional help. This case report describes a 12-year-old girl with recurrent oral ulcers, severe chronic periodontitis, and pathological tooth migration as the initial and main clinical symptoms of CyN. Two novel mutations in *ELANE*, c.180T>G (p.I60M) and c.182C>G (p.A61G) associated with CyN were observed. Bioinformatics research indicated lower stability and impaired molecular linkages of the mutant neutrophil elastase (NE) encoded by *ELANE*. However, the enzyme affinity to the classic substrate Suc–Ala–Ala–Ala–pNA was not substantially changed, suggesting that the impaired integrity and stability of the mutant NE, rather than catalytic deficiency, might be the pathogenic mechanism of *ELANE* mutation-induced neutropenia. The patient was prescribed scaling and root planing (SRP) and monthly periodontal maintenance without systemic management. Although the routine periodontal treatment was occasionally interrupted by the 2019 coronavirus pandemic, her periodontal devastation remained well-remitted in the 5-year follow-up assessment. The results of this study confirmed the importance of plaque control and proper diagnosis in the periodontal management of such patients and provide better clinical references. In addition, the novel mutations identified in this study expand the spectrum of known *ELANE* mutations in CyN and further contribute to knowledge regarding its pathogenic mechanism.

## Introduction

Cyclic neutropenia (CyN) is a rare hematological disease characterized by recurrent peripheral neutropenia involving cycles with 21-day intervals (Dale and Hammond, 1988; Dale et al., 2000). Associated symptoms such as fever, malaise, headaches, respiratory infections, and abdominal discomfort commonly accompany this condition (Lakshman and Finn, 2001). Each episode lasts for 3–6 days, and the affected individuals are generally normal between bouts. The predictable cyclicity is always used in differential diagnosis with severe congenital neutropenia (SCN), which manifests as more serious and sustained neutropenia (Horwitz et al., 2013; Skokowa et al., 2017). A total of 80–100% of CyN and 35–63% of SCN cases are caused by heterozygous mutations in *ELANE*, which maps to chromosome 19p13.3 and encodes neutrophil elastase (NE). Aberrant NE is thought to accelerate the apoptosis of maturing neutrophil precursors, subsequently decreasing the peripheral neutrophil count (Nanua et al., 2011; Walter and Ron, 2011; Borregaard, 2014; Rydzynska et al., 2021). The pathogenic genetic variants of CyN and SCN differ and the clinical presentations vary according to the mutations. In addition, age, hormones, and environmental factors may also contribute to the corresponding clinical manifestations.

Patients with CyN always exhibit oral manifestations, including recurrent aphthous ulcers (RAU), gingivitis, severe periodontitis, and the concomitant inflammatory mediator increases (Ródenas et al., 1992; Isola et al., 2020). Oral manifestations may be the first symptom in patients with CyN and may easily be misdiagnosed in dental clinics. In addition, these symptoms usually occur around early childhood and persist after adolescence, leading to a premature loss of permanent teeth and poor oral function. Therefore, early diagnosis and management of this complicated condition are challenging but essential. Herein, we report the case of a 12-year-old girl affected by CyN with oral manifestation (RAU and advanced periodontal destruction) as the first and sole symptom. This case presented two spontaneous mutations in exon2 of *ELANE*, c.180T>G (p.I60M) and c.182C>G (p.A61G), not previously reported in CyN. Scaling and root planing (SRP) along with monthly periodontal maintenance were performed. After 5 years of follow-up, the patient showed retardation of periodontal decline, with no tooth loss and little alveolar bone absorption. This case expands the spectrum of known mutations of congenital neutropenia, contributes new insights into the pathological mechanism of congenital neutropenia, verifies the importance of early diagnosis and plaque control, and summarizes our experiences.

## Case report

A 12-year-old Chinese girl visited Shenzhen People’s Hospital with a chief complaint of tooth displacement. Her parents had noticed tooth mobility and migration, red and swollen gums, and recurrent ulcers for the past 4 years.

This patient had had RAU since 4 years of age. After she reached menarche at 11 years, the RAU showed exacerbation during her menstrual period. Her general growth was adequate for her age, and she had a healthy mental and physical status, except for the oral symptoms. At 18 days of age, the patient developed hyperpyrexia and pneumonia after *Bacillus* Calmette-Guerin vaccination. Before 2 years of age, the patient had shown occasional skin pustulosis. She was diagnosed with hyper-IgE syndrome at 2 years of age and was administered thymopeptide for 6 months until her IgE levels returned to normal. During treatment, the doctors once noted a decrease in white blood cell counts but did not recommend further treatment. Similar symptoms were not observed in any of the three generations of her family. The patient’s parents and younger sister were healthy and without immunological diseases. The patient brushed her teeth twice daily, 1–2 min each time, in a horizontal brushing motion, without using dentin floss or an interproximal brush, and had no bad habits.

**FIGURE 1:**
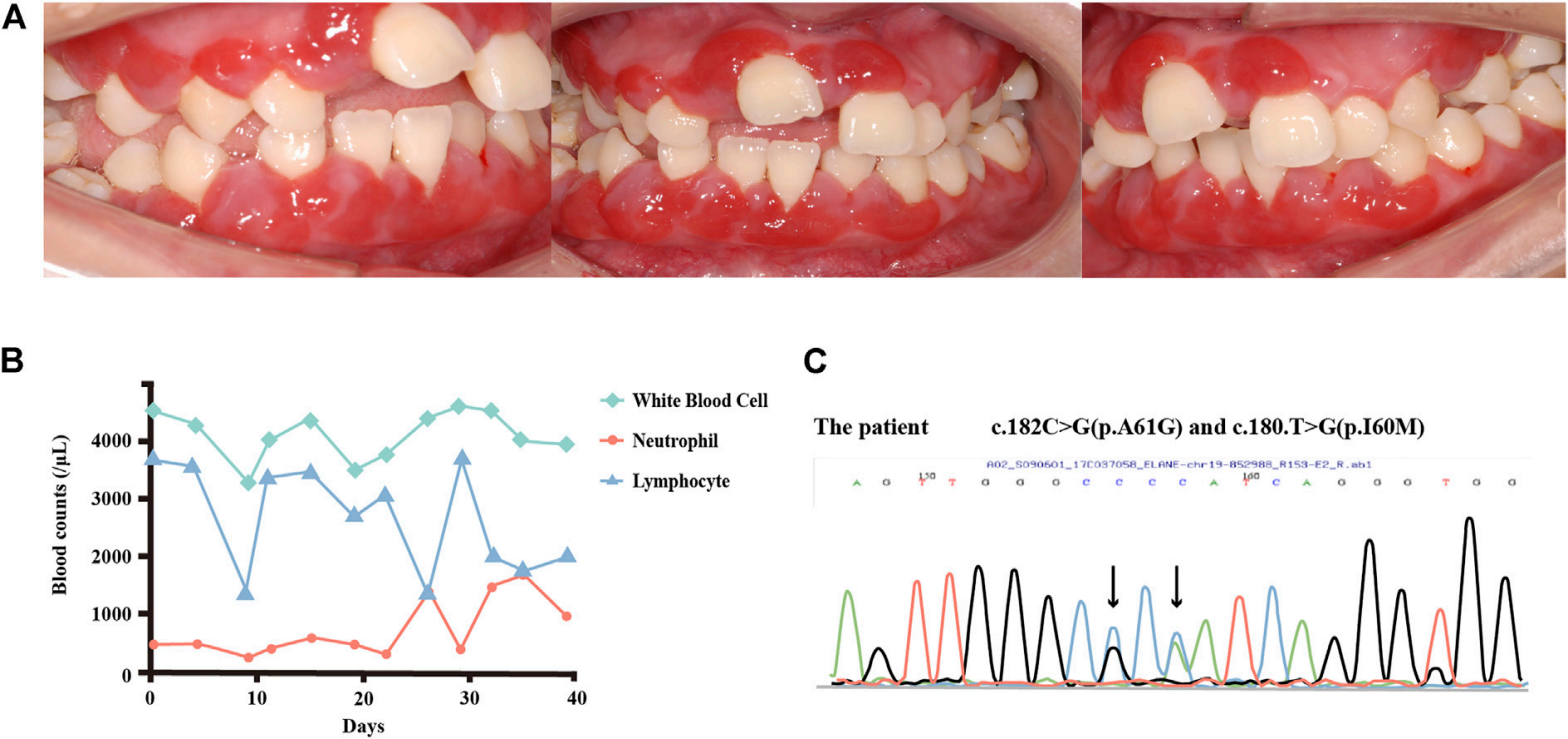
**(A)** Intraoral view of the patient in 2017. The marginal and attached gingiva were fiery red, with swelling and bleeding on contact. **(B)** Changes in the white blood cell, neutrophil, and lymphocyte counts over 6 weeks. The patient’s neutrophil counts appeared to cycle between 200/μL and 1,500/μL, with the lymphocyte counts showing an inverse cycle pattern. **(C)** DNA sequence analysis of *ELANE*. The patient carries two spontaneous mutations. As both forward and reverse sequences can be used for verification in Sanger sequencing, the bases in the peak diagram may be the reverse complementary sequence of the detected bases. For instance, in c.180T > G, T > G is shown as A > C in the peak diagram.

The initial examination revealed that the marginal and attached gingiva was fiery red, swollen, and bled on contact (Figure 1A). The periodontal examination revealed general and deep periodontal pockets (6–9 mm). Teeth 16, 14, and 41 showed class III mobility, while teeth 21, 24, 25, 32, and 36 showed class II mobility. The patient also showed tooth migration and malocclusion, as well as poor oral hygiene. As the severe bone loss was not consistent with the dental plaque accumulation, further investigation for hematological disorders was initiated. A severe decrease in neutrophil count (280/μL), mildly decreased peripheral leukocyte count (3,340/μL), and mildly increased monocyte count (1,280/μL) were observed. The lymphocyte, eosinophil, and basophil counts were within normal limits (Supplementary Table S1). However, the peripheral smear results showed a severe decrease in segmented neutrocytes (2%), an increased monocyte proportion (41%), as well as mildly increased eosinophilic granulocytes (11%), basophilic granulocytes (2%), and lymphocytes (44%). The morphology and quantity of the erythrocytes and blood platelets were normal. Therefore, the patient was diagnosed with neutropenia. Combined with the early onset of symptoms and long course of recurring inflammation, the tentative diagnosis was congenital neutropenia. Antibody screening for anti-neutrophil cytoplasmic protease antibody PR3-ANCA (-), and anti-neutrophil cytoplasmic myeloperoxidase antibody (-) were performed to preclude autoimmune diseases (Supplementary Table S3). PCR screening for Epstein–Barr virus DNA (EBV-DNA) (-) and human cytomegalovirus DNA (HCMV-DNA) (-) was used to eliminate infectious diseases (Supplementary Table S3). The patient’s lack of special drug use and toxic substance exposure further excluded other potential acquired neutropenia. Her phosphorus level was slightly elevated (1.7 mmol/L) and her serum potassium, sodium, chloride, and calcium levels were within normal limits (Supplementary Table S2). Her CD3^+^CD4^+^(23.6%) and CD4^+^/CD8^+^Th/Ts (0.54%) levels were lower, while her IgG1 (17.40 g/L), IgG2 (7.85 g/L), and IgG3 (1.21 g/L) concentrations were above the normal limits (Supplementary Table S2). All other clinical findings are shown in Supplementary Table S4.

DNA sequencing was performed of samples from the patient and her parents to further confirm the diagnosis. No *ELANE* mutations were detected in the samples from her parents. Mutational analyses of the patient’s sample revealed two substitutions in the 180th base (T to G), causing the amino acid substitution p.I60M, and in the 182nd base (C to G), causing the amino acid substitution p.A61G in exon2 (Figure 1C). These mutations were previously reported in patients with SCN (Xia et al., 2009) but had not yet been related to the cyclicity characteristics of CyN. The patient was then referred to Shenzhen Children’s Hospital, where blood tests were conducted over 6 weeks. The evaluations of neutrophil count revealed a slight fluctuation between 200–500/μL during the first 3 weeks, which increased to 1,500/μL on the 25th day, before starting the following cycle with a greater amplitude (Figure 1B). An inverse trend in lymphocyte counts was observed, along with an oscillatory pattern in neutrophil count. A timeline with relevant examination, diagnosis, and treatment information is presented in Supplementary Figure S2.

**FIGURE 2:**
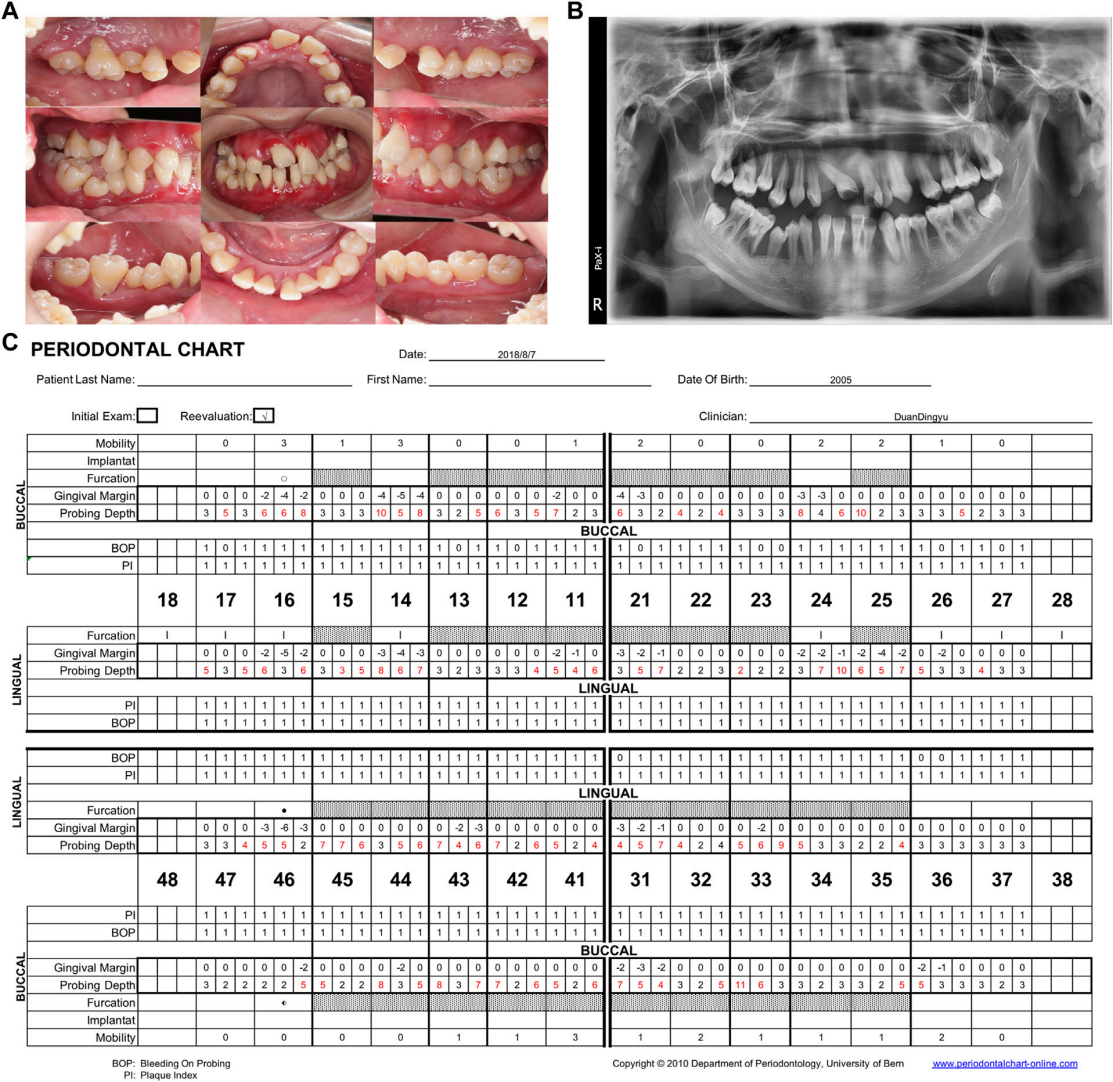
Follow-up examination in 2018. **(A)** Intraoral view showing redness, gingiva swelling and recession, irregular tooth alignment, and serious occlusion disorder. **(B)** Panoramic radiograph showing general disappearance of the lamina dura and complete horizontal absorption of the alveolar bone to 1/2–2/3 of the root length. The alveolar bone loss of 14, 16, 24, 25, and 46 involved the apical roots. **(C)** Periodontal chart. 93% bleeding on probing, mean PD: 4.2 mm. PD, probing depth.

After the final diagnosis of CyN, RAU, and periodontitis associated with systemic disease, the patient was referred to West China Hospital at Sichuan University for treatment. The periodontal examination revealed extensive and deep periodontal pockets, with depths reaching 6–10 mm around teeth 16, 14, 12, 11, 21, 24, 25, 31, 33, and 41–45. Severe gingival recessions of 4–7 mm were recorded at teeth 16, 14, 21, 25, and 46. Superficial dentin caries was observed on the occlusal surfaces of teeth 37 and 47 (Figures 2A,C). Panoramic radiography revealed generalized severe horizontal bone loss (Figure 2B). Obvious bone absorption was noted in the furcation areas of teeth 16 and 46. The recommended treatment plan was as follows:

1. Oral hygiene instructions (OHI),
2. Extraction of tooth 14,
3. SRP and local administration of minocycline ointment,
4. Resin filling restorations for teeth 37 and 47,
5. Periodontal maintenance before each menstruation,
6. Extraction of hopeless teeth at age 18 years,
8. Prosthetic rehabilitation.

The initial periodontal therapy included SRP, education, pharmacology, self-care, and occlusal adjustment. We performed careful periodontal maintenance every month before each menstruation. Minocycline ointment (Periocline; Sunstar Co., Japan) was applied to the periodontal pockets.

Since the patient was clinically well, without major systemic infections or complications, we did not recommend systemic administration following a comprehensive assessment of the risks and benefits. However, in January 2022, the patient developed a fever and was admitted to the hospital, where she received piperacillin–tazobactam, oseltamivir, and granulocyte colony-stimulating factor (G-CSF) therapy. As shown in Supplementary Figure S1, her neutrophil and WBC counts increased to normal after 3 days of G-CSF administration. The RAU was relieved after G-CSF therapy and antibiotic treatment.

**FIGURE 3:**
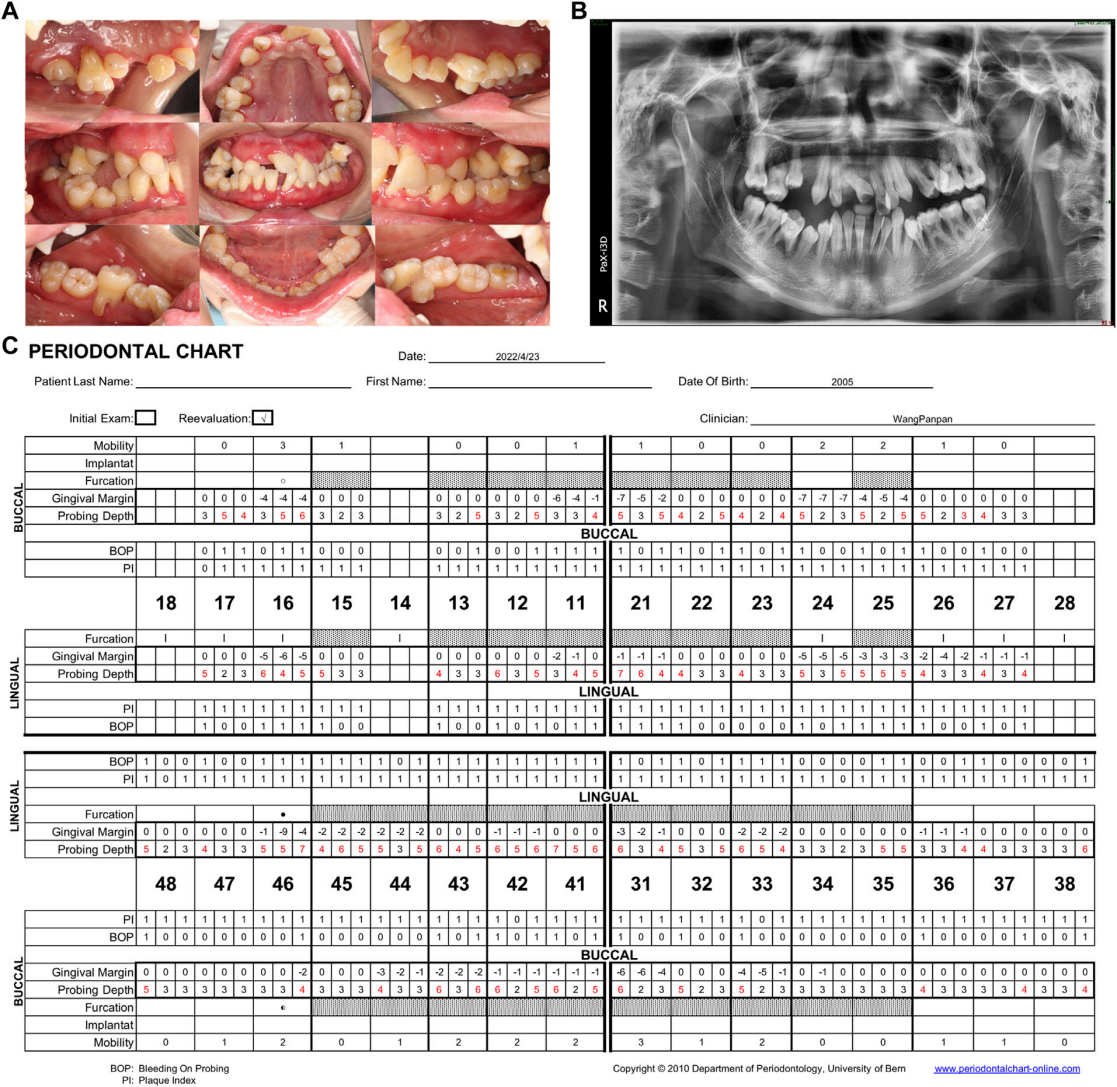
**(A)** Intraoral view in the Hospital of Stomatology, Sun-Yat sen University in 2022. After 5 years of follow-up, the gingival swelling and redness were reduced. **(B)** Panoramic radiograph showing general horizontal bone loss of 1/3–2/3 of the root length. **(C)** Periodontal chart. Specialized periodontal examination record of the patient after the 5-year follow-up. 50% bleeding on probing, mean PD: 3.9 mm. PD, probing depth.

After 5 years of periodontal follow-up, no extra teeth were lost, and the progression of periodontitis showed a degree of retardation. In contrast with the first visit in 2018, the bleeding on probing (50% in 2022 *versus* 93% in 2018) had decreased and the probing depth (mean PD: 3.9 mm in 2022 *versus* mean PD: 4.2 mm in 2018) had lessened considerably (Figure 3).

**FIGURE 4:**
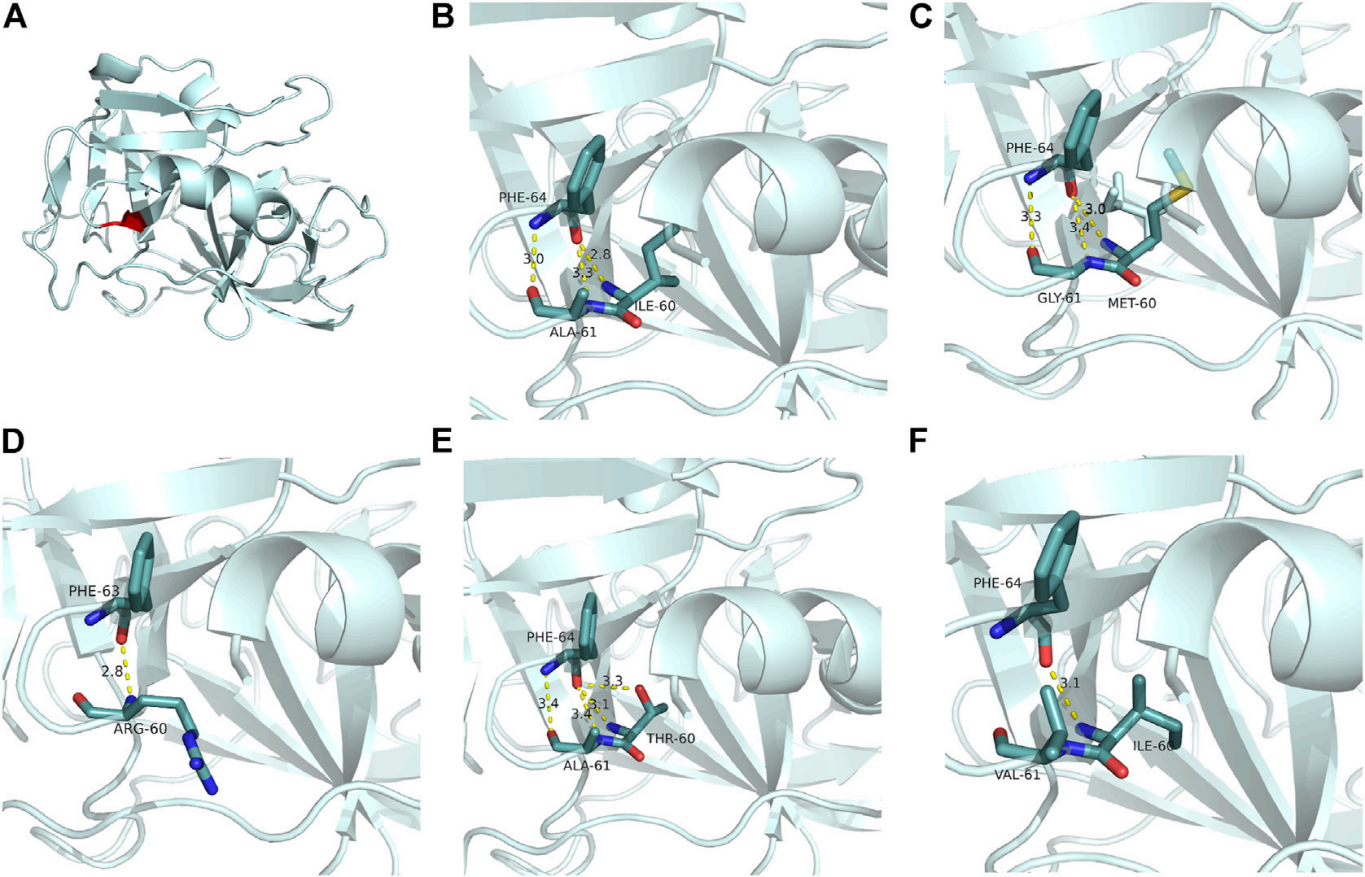
Ribbon diagrams of neutrophil elastase. **(A)** Position (red) of residues Ile60 and Ala61. **(B)** H-bonds between residues Ile60 and Ala61 and their substrate Phe64. **(C)** Changed H-bonds in NE mutant I60M, A61G (the present case). While three H-bonds remained between residues 60 and 61 and Phe64, the bond lengths increased, indicating weakened connections. **(D)** Phe64 lost two H-bonds with Arg60. (SCN, with a synchronous mutation of GFI1). **(E)** Phe64 obtained an additional H-bond with Thr60 (SCN). **(F)** Phe64 lost two H-bonds with Val60 (overlapping between SCN and CyN). Partial tertiary structures were deleted to show the variants and adjacent residues in the ribbon diagrams. The color codes for the molecules and bonds are as follows: carbon, pale cyan; oxygen, red; nitrogen, blue; sulfur, yellow; H-bond, yellow. PD, probing depth.

We then established a predictable tertiary structure of mutational neutrophil elastase through SWISS-MODEL homologous modeling (https://swissmodel.expasy.org/) and further characterized the NE modification using bioinformatics approaches. NE with the protein database bank ID 1b0f was used as the reference structure. The intra-molecular interactions around the mutant amino acids are depicted in Figure 4. As the ribbon diagram shows, the Ile60 and Ala61 residues are located at the beta-sheet and beta-turn areas, respectively. They are connected to Phe64 by three hydrogen bonds in wild-type NE. In the NE mutant, the three H-bonds between the 60th, 61st, and phe64 remained, but the bond lengths had increased, indicating weakened connections. Mutational energy calculations performed using the Discovery Studio server with a CHARMm force field revealed a dramatic reduction in stability of the double-mutated amino acid sequence, with a mutation energy of +4.18 kcal/mol. We also used Autodock Vina to predict the change in NE catalytic performance according to NE active pocket affinity with the classical substrate Suc–Ala–Ala–Ala–pNA. However, we observed no significant difference between the wild-type and mutant NE.

## Discussion

Timely and accurate diagnosis is important for CyN patients. Oral manifestations often occur as the first symptom of immunodeficiency diseases, and those patients are easily misdiagnosed as simple RAU or aggressive periodontitis without careful differentiation.

The patient in this case sought medical treatment with a chief complaint of tooth mobility and migration, red and swollen gums, and recurrent ulcers lasting for 4 years. The differential diagnosis was performed sequentially. First, we clarified whether the RAU lesion was caused by localized mucosal ulcerative diseases like erythema multiforme, or by infections or specific systemic diseases such as herpesvirus infection, HIV infection, Behcet’s syndrome, Sweet’s syndrome, and leukemias. Considering the unpredictable recurrence of oral ulcers and the accompanying systemic symptoms, the patient’s RAU lesion was diagnosed as “aphthous-like ulcers with systemic disease”. (Scully, 2006) Second, based on the discordance of the alveolar bone destruction and severity of dental plaque, we diagnosed the patient with “periodontitis associated with systemic disease” (Scully, 2006). Third, a significant reduction in neutrophil count (280/μL) was observed in the blood tests. The early onset of symptoms, long-term and recurring inflammation, lack of history of special drug use, lack of exposure to toxic and harmful substances, and negative results of specific antibody examinations and virus tests all pointed to congenital neutropenia. The 25-day oscillation in neutrophil counts in this case and lack of extra-hematopoietic manifestation suggested a diagnosis of CyN. DNA sequencing analysis further genetically confirmed the diagnosis.

Timely diagnosis and early treatment initiation are crucial to stop the disease progression at an early stage. CyN is a rare disease with an incidence of 0.5–1 case per million (Wright et al., 1981). However, the early diagnosis of CyN is difficult because of the need for serial differential blood count monitoring of absolute neutrophil count (2–3 times per week for 6–8 weeks) to determine the oscillatory pattern of neutrophil count. In children presenting with severe periodontitis, systemic problems should not be ruled out without sufficient testing. Dentists should review the patient’s medical status thoroughly, analyze differential diagnoses, and make appropriate referrals.

Understanding the interplay between *ELANE* mutations and clinical manifestations is important. A total of 80–100% of CyN and 35–63% of SCN cases present with *ELANE* mutations (Horwitz et al., 1999; Horwitz et al., 2007). Among reported *ELANE* mutations, 88% exist solely in CyN or SCN, while 12% exist in both diseases (overlapping mutations) (Horwitz et al., 1999). The patient in the present case showed two novel concurrent spontaneous *ELANE* mutations: c.180T>G (p.I60M) and c.182C>G (p.A61G). Both have been reported in SCN (Germeshausen et al., 2013) but not in CyN (in the gnomAD UCSC Genome Browser). These two extra overlapping mutations might cause both diseases. Determining the genotype-phenotype correlation is clinically significant. For instance, mutations only observed in CyN or overlapping mutations are more strongly associated with oral ulcers (96 of 99 patients, 97%) and are less likely to develop pneumonia (36 of 99 patients, 36%) compared to those unique to SCN (61 of 97 patients, 63%, both) (Makaryan et al., 2015). The clinical manifestations observed in the present case were consistent with this pattern.

The present case provided insights into the H-bonds among several mutant NEs and help explain the pathological mechanism of *ELANE*-related neutropenia. We found that I60M and A61G substitutions did not change the number of H-bonds but rather reduced their connections. This decreased energy due to the mutations also indicated a decreased stability of the mutant NE, consistent with the hypothesis that *ELANE* mutations result in the production of misfolded proteins to induce an unfolded protein response and apoptosis. Combined with the nearly identical NE affinities to the classical substrate before and after mutation, we suggest that protein integrity and stability instead of catalytic defects might be the mechanism by which *ELANE* mutations contribute to neutropenia.

However, the effect of changes in NE intramolecular interactions (such as H-bonds) on clinical phenotypes requires further investigation. Our analysis of four reported mutations in Ile60 or Ala61 showed that most mutations in SCN changed the number of H-bonds. For instance, the p.I60T mutation (Dale et al., 2000; Rydzynska et al., 2021) gained an additional H-bond with Thr60 (Figure 4E), while the p.A61V and p.I60_A61delinsR mutations lost two H-bonds (Figures 4D,F) (Horwitz et al., 1999; Dale et al., 2000; Germeshausen et al., 2013; Rydzynska et al., 2021). In the present case, the number of bonds remained the same (three), but they became weaker (Figure 4C). The two substituted amino acids, Met60 and Gly61, have basic properties very similar to those of the original amino acids, including size, side-chain structure, isoelectric point (I-M:6.02 to 5.74, A-G:6.0 to 5.97), and polarity (all hydrophobic except for Gly, which had weak polarity). Therefore, the intra-molecular change in NE in the present case was so slight that the oral manifestation was the sole symptom.

Furthermore, there remains controversy regarding the mechanism of overlapping mutations and influencing factors. *ELANE* mutations seem to be a prerequisite for neutropenia, while the patient episodes, clinical features, and systemic symptoms are dependent on comprehensive complicated factors such as hormones and age, or other gene modifiers like GFI1 and LEF1 (Germeshausen et al., 2001; Xia et al., 2009; Newburger et al., 2010; Cho and Jeon, 2014; Aota et al., 2019; Mir et al., 2020). In the present case, the appearance of RAU and neutropenia was closely related to menstruation; however, the cause requires further investigation.

Considering the systemic factors involved, dental plaque management is essential. Most patients with CyN with chronic complications or infections experience tooth loss (Wright et al., 1981). A registry-based study of 45 patients in France under 30 years of age reported that, if plaque control was not emphasized, 18 and 17 showed partial and complete edentulism, respectively (Wright et al., 1981). In contrast, regular nonsurgical periodontal treatment has been proven to reduce dental plaques and improve clinical outcomes. (Gorlin and Chaudhry, 1960; Pernu et al., 1996; Hastürk et al., 1998; da Fonseca and Fontes, 2000; Goultschin et al., 2000; Block et al., 2015). Therefore, in cases with timely diagnosis, better periodontal outcomes were more likely in patients receiving regular periodontal and antibiotic treatments (Pernu et al., 1996; Hastürk et al., 1998; Goultschin et al., 2000). Patients who did not receive proper treatment showed worsened conditions, including tooth extraction and severe bone loss (Ródenas et al., 1992; Dale et al., 2000; Horwitz et al., 2007; Skokowa et al., 2017). Thus, professional prophylaxis and scaling should be performed monthly and even weekly when the neutrophil count starts to decline (Aota et al., 2019). The patient and her caregivers in the present case were educated regarding the importance of oral hygiene and trained to provide adequate home care, including correct brushing, flossing, and waterpik use, with chlorhexidine rinses as an adjunct (Lu and Meng, 2012). The use of fluoride rinse or gel could be additionally considered because of the high morbidity of caries.

The patient fully understood her state of illness and showed good medical compliance. Her supervisors also emphasized periodontal management, and both pushed through difficulties in adhering to monthly clinical visits. However, since the coronavirus disease outbreak in 2019, the patient’s periodontal treatment has been interrupted occasionally. During the local epidemic, we had to turn to online guidance and supervision on the effects of oral hygiene control. Despite these difficulties, her high compliance and regular periodontal treatment led to a good outcome with no extraction.

This patient visited the dentist with severe tooth migration, which was aggravating every year. Specifically, tooth 31 presented with severe mobility and had notable absorption of the mesial alveolar bone and broadening of the periodontal space owing to occlusal trauma. In such cases, further dental treatment is important, as tooth displacement can make oral hygiene practice difficult, thus promoting a pathologic environment conducive to more severe periodontitis. Progressive periodontitis might aggravate tooth migration, potentially leading to a vicious circle; thus, the necessity for earlier interventions to ameliorate this cycle warrants further discussion. A periodontal splint might have been considered earlier in this case, as it is easy to operate and the prognosis is relatively controllable, even though it cannot correct the deflection to normal. Although periodontal splinting fixation might have interfered with alveolar bone growth, it might have reduced occlusal trauma, with subsequent follow-up performed to determine whether to continue its use based on the findings.

Other multi-disciplinary treatment options for this patient were also discussed. Although the patient responded well to G-CSF, systemic therapy was not prescribed by her physician unless major systemic symptoms appeared. Combined with bone loss, orthodontic treatment should be avoided because there is an increased risk of accelerated periodontal breakdown. Periodontal flap surgery was not recommended because of the high potential for infection. We plan to extract hopeless teeth, such as 16, 15, 31, and 46, when the patient reaches maturity, at which time occlusal reconstruction with a periodontal splint will be recommended. Block et al. (2015) reported a case of eight implants in a 59-year-old patient with CyN, in which the 3-year follow-up revealed a stable alveolar bone level. Therefore, if the patient in the present case loses more teeth, a dental implant could be placed in the future.

In conclusion, we described a case of CyN with RAU and severe periodontitis as the main manifestations. Two novel spontaneous *ELANE* mutations were identified. First, this case demonstrated that *ELANE* mutations are a prerequisite for neutropenia and that the clinical features depend on comprehensive factors. Second, the manifestation of RAU and periodontitis could be early diagnostic signatures of systemic diseases such as CyN, which require a timely and accurate diagnosis for positive outcomes. Third, the proper management of dental plaques can effectively relieve gingival inflammation and slow alveolar bone loss.

## Data Availability

The datasets for this article are not publicly available due to concerns regarding participant/patient anonymity. Requests to access the datasets should be directed to the corresponding authors.
