# Supplementary material for: Case report: Five-year periodontal management of a patient with two novel mutation sites in ELANE-induced cyclic neutropenia
Source: Front Genet. 2022 Nov 1;13:972598. doi: 10.3389/fgene.2022.972598 (PMC9663835; doi:10.3389/fgene.2022.972598)
Supplement: Supplementary file 1 [file DataSheet1.docx]

Supplementary Material

# 1 Supplementary Data

## 1. 1 Supplementary Figure

**Supplementary Figure 1.**Changes in the WBC, neutrophils, lymphocytes and monocytes before and after administration of G-CSF in 2022 Jan. The patient's neutrophil counts and WBC counts climbed up to 4220/μL and 9010/μL, reaching the normal level after using G-CSF continuously for 3 days.


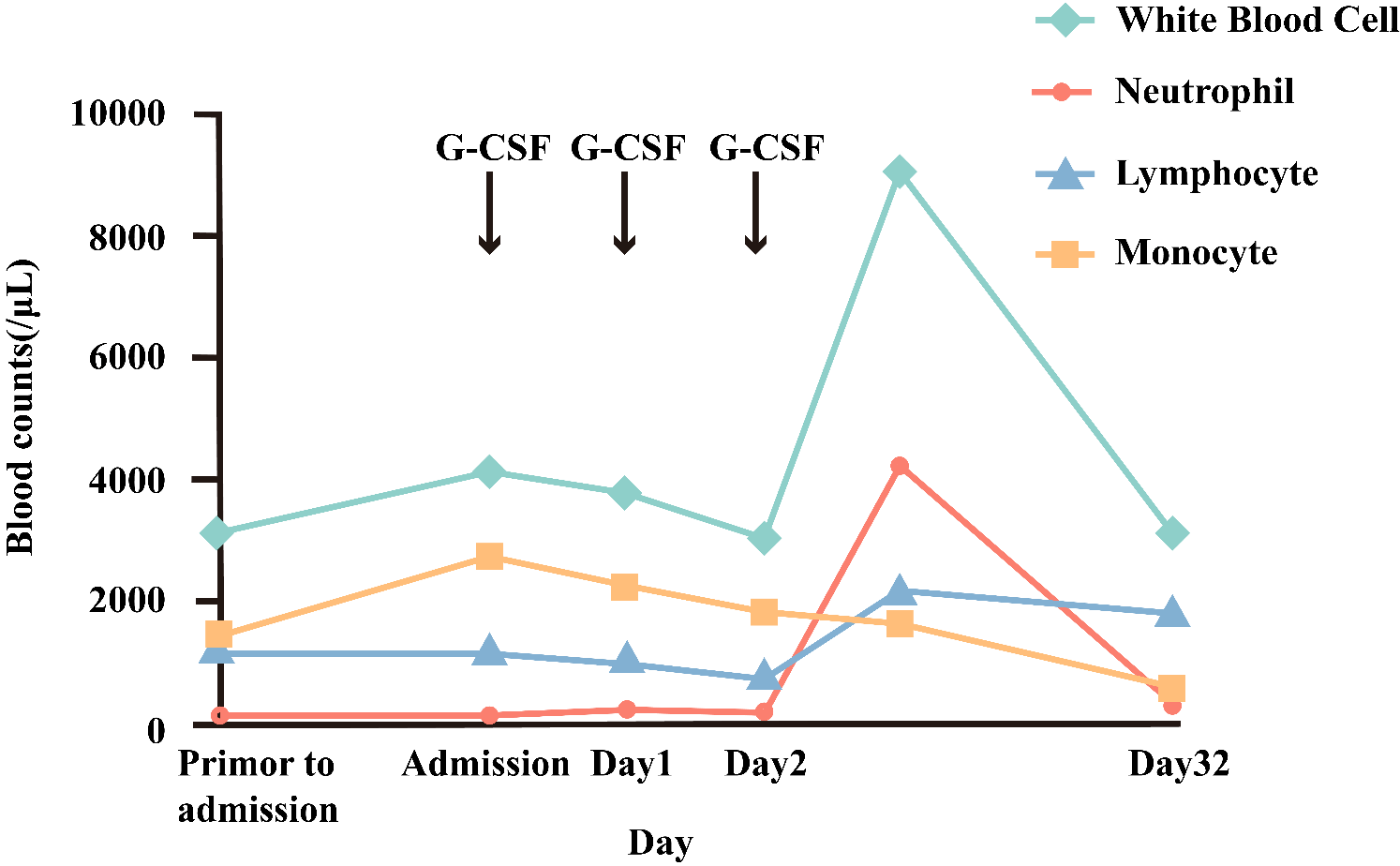


**Supplementary Figure 2.** A timeline of relevant examination, diagnosis and treatment.


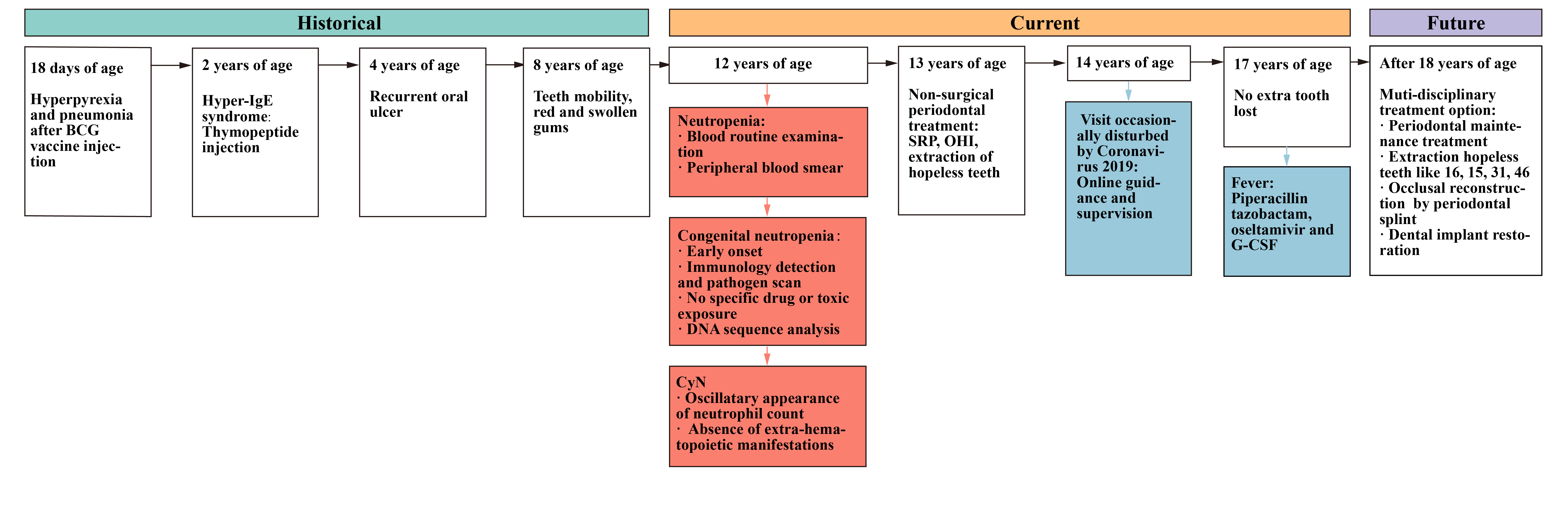


## 1.2 Supplementary Tables

**Supplementary Table 1**.

The patient's hematology test showed a severe lack of neutrophil count, a mildly decreased peripheral leukocyte count, and a mildly increased monocyte count.

| Item | Result | Unit | Range |  |
| --- | --- | --- | --- | --- |
| White Blood Cell | 3340 | /μL | 4000-10000 | ↓ |
| Neutrophil | 280 | /μL | 2000-7500 | ↓↓ |
| Lymphocyte | 1400 | /μL | 800-400 |  |
| Monocyte | 1280 | /μL | 120-800 | ↑ |
| Eosinophil | 340 | /μL | 0-500 |  |
| Basophil | 40 | /μL | 0-100 |  |
| Red Blood Cell | 5580 | /μL | 3500-5000 | ↑ |
| Hemoglobin(Hb) | 135 | g/L | 110-150 |  |
| Hematocrit(HCT) | 43.4 | % | 37-43 | ↑ |
| Mean Corpuscular Volume(MCV) | 77.8 | f1 | 82-92 | ↓ |
| Mean Corpuscular Hemoglobin(MCH) | 24.2 | pg | 27.0-31.0 | ↓ |
| Platelet(PLT) | 376 | ×10^3/μL | 100-300 | ↑ |
|  |  |  |  |  |

**Supplementary Table 2.**

The patient’s immunoglobulin and complement test, lymphocyte and subset test, electrolyte composition analysis and immunoglobin IGG subtype determination.

|  |  |  |  |  |
| --- | --- | --- | --- | --- |
| Item | Result | Unit | Range |  |
| **Immunoglobulin and complement** | |  |  |  |
| complement C3 | 1.54 | g/L | 0.80-1.81 |  |
| complement C4 | 0.50 | g/L | 0.15-0.57 |  |
| ImmunoglobulinA | 3.55 | g/L | 0.70-4.5 |  |
| ImmunoglobulinG | 26.71 | g/L | 8-18 | ↑ |
| ImmunoglobulinM | 1.14 | g/L | 0.40-2.5 |  |
| Immunoglobulin E | 47.04 | IU/ml | 0.1-150 |  |
| **Lymphocyte Subsets** |  |  |  |  |
| CD3+T cell(CD3+T) | 76.2 | % | 50-84 |  |
| CD3+CD4+helper T cell(CD3+CD4+) | 23.6 | % | 27-51 | ↓ |
| CD3+CD8+cytotoxic T cell(CD3+CD8+) | 43.5 | % | 15-44 |  |
| CD3-CD19+ B cell(CD3-CD19+) | 11 | % | 5-18 |  |
| CD3-CD16+56+ NK cell(CD3-CD16+56+) | 12.1 | % | 7-40 |  |
| CD4+/CD8+ Th/Ts ratio | 0.54 | % | 0.68-2.4 | ↓ |
| **Electrolyte composition analysis** | |  |  |  |
| K | 4.8 | mmol/L | 3.5-5.5 |  |
| Na | 137.8 | mmol/L | 135-145 |  |
| Cl | 101.0 | mmol/L | 96-110 |  |
| Ca | 2.4 | mmol/L | 2.05-2.55 |  |
| P | 1.7 | mmol/L | 0.8-1.5 | ↑ |
| carbon dioxide combining power ( CO2-CP ) | 28.5 | mmol/L | 22-34 |  |
| **Immunoglobulin IGG subtype determination** | | |  |  |
| IgG1 | 17.40 | g/L | 4.9-11.4 | ↑ |
| IgG2 | 7.85 | g/L | 1.5-6.4 | ↑ |
| IgG3 | 1.21 | g/L | 0.11-0.85 | ↑ |
| IgG4 | 1.60 | g/L | 0.03-2 |  |
|  |  |  |  |  |

**Supplementary Table 3.**

Other analyses of autoimmune molecular signature

|  |  |  |  |
| --- | --- | --- | --- |
| Item | Result | Unit | Normal Range |
| EBV-DNA | <1.0×10^3 | copies/ml | <1.0×10^3 |
| HCMV-DNA | <1.0×10^3 | copies/ml | <1.0×10^3 |
| HbsAg | 0.00 | IU/ml | <0.05 |
| HCV antibody | 0.09 | S/CO | <1.0 |
| HIV-1/-2 antibody | 0.08 | S/CO | <1.0 |
| Syphilis specific antibody | 0.07 | S/CO | <1.0 |
| Syphilis serotonin | - |  | - |
| Anti-neutrophil cytoplasmic protease antibody(PR3-ANCA) | 2.173 | U/ml | 0.000-15.000 |
| Anti - neutrophil cytoplasmic myeloperoxidase antibody(MPO-ANCA) | 0.929 | U/ml | 0.000-15.000 |
| Anti-nuclear antibody (ANA) | - |  | - |
| Anti-dsDNA antibody | - |  | - |
| Anti-ribonucleoprotein antibody | - |  | - |
| Anti-SCL-70 antibody | - |  | - |
| Anti-Jo-1 antibody | - |  | - |
| Anti-Rib antibody | - |  | - |
| C-reactive protein(CRP) | 18.6 | mg/L | <5 |
| Rheumatoid Factor(RF) | <10.1 | IU/ml | <20 |
| Circulating immune complex (CIC) | 0.08 | 0.D | <0.15 |
| Antistreptolysin-O(ASO) | <50.6 | IU/ml | <200 |
| Anti-streptococcus DNAase B(ADNaseB) | 85 | U/ml | <200 |
| Anti-SSA antibody | - |  | - |
| Anti-SSB antibody | - |  | - |
| Anti-Sm antibody | - |  | - |
|  |  |  |  |

**Supplementary Table 4.** Other obtained physical examination (PE) and clinical findings

|  |  |  |  |  |
| --- | --- | --- | --- | --- |
| Item | Result | Unit | Range |  |
| **Minor elements** | |  |  |  |
| Zn | 72.94 | μmol/L | 43-229 |  |
| Fe | 5.65 | mmol/L | 4.2-11.8 |  |
| Ca | 1.61 | mmol/L | 1.1-2.5 |  |
| Cu | 15.24 | μmol/L | 9.0-35 |  |
| Mg | 1.32 | mmol/L | 0.6-2.0 | ↑ |
| Cd | <0.50 | μg/L | 0-7.4 |  |
| Pb | 15.08 | μg/L | 0-100 |  |
| **Other hematological data** |  |  |  |  |
| Indirect bilirubin | 5.30 | μmol/L | 1.7-16 |  |
| Globulin | 48 | g/L | 20-40 | ↑ |
| Fasting blood-glucose | 5.32 | mmol/L | 3.9-6.1 |  |
| Triglyceride | 0.87 | mmol/L | 0.4-2.3 |  |
| Total cholesterol | 3.85 | mmol/L | 3.4-6.5 |  |
| High-density lipoprotein | 1.13 | mmol/L | 0.9-1.91 |  |
| Low-density lipoprotein | 2.12 | mmol/L | 2.08-4.14 |  |
| Blood uric acid | 273 | μmol/L | 90-420 |  |
| **Renal function analysis** | |  |  |  |
| Urea nitrogen | 2.41 | mmol/L | 2.5-7.5 | ↓ |
| Creatinine | 47.2 | μmol/L | 44-133 |  |
| **Hepatic function analysis** | | |  |  |
| Glutamic-pyruvic transaminase | 9 | U/L | 0-40 |  |
| Glutamic oxalacetic transaminase | 13 | U/L | 0-45 |  |
| Alkaline phosphatase (ALP) | 185 | U/L | 15-121 | ↑ |
| Total protein (TP) | 91.4 | g/L | 60-83 | ↑ |
| Serum albumin | 43.3 | g/L | 35-55 |  |
| Aspartate aminotransferase mitochondrial isoenzyme | 4.7 | U/L | <15 |  |
| Γ-glutamyl transpeptidase | 12 | U/L | 0-60 |  |
| Total bilirubin | 6.6 | μmol/L | 1.7-20 |  |
| Direct bilirubin | 1.3 | μmol/L | 0-6 |  |
| Prothrombin time (PT) | 11.70 | s | 9-13 |  |
| International normalized ratio (INR) | 1.02 | - | 0.8-1.5 |  |
| Fibrinogen | 4.28 | g/L | 2-4 | ↑ |
| Activated partial thromboplastin time (APTT) | 26.40 | s | 20-40 |  |
| Thrombin time | 17.20 | s | 14-21 |  |
|  |  |  |  |  |
